# Improved Surface-Enhanced Raman Scattering Properties of ZrO_2_ Nanoparticles by Zn Doping

**DOI:** 10.3390/nano9070983

**Published:** 2019-07-06

**Authors:** Peng Ji, Zhu Mao, Zhe Wang, Xiangxin Xue, Yu Zhang, Jiaao Lv, Xiumin Shi

**Affiliations:** 1 College of Chemical Engineering, Changchun University of Technology, Changchun 130012, China; 2 School of Chemistry and Life Science, Changchun University of Technology, Changchun 130012, China; 3 Key Laboratory of Preparation and Applications of Environmental Friendly Materials, Jilin Normal University, Changchun 130103, China

**Keywords:** Zn-doped, ZrO_2_, SERS, charge transfer, enhancement factor

## Abstract

In this study, ZrO_2_ and Zn–ZrO_2_ nanoparticles (NPs) with a series of Zn ion doping amounts were synthesized by the sol-gel process and utilized as substrates for surface-enhanced Raman scattering (SERS). After absorbing the probing molecule 4–mercaptobenzoic acid, the SERS signal intensities of Zn–ZrO_2_ NPs were all greater than that of the pure ZrO_2_. The 1% Zn doping concentration ZrO_2_ NPs exhibited the highest SERS enhancement, with an enhancement factor (EF) value of up to 10^4^. X-ray diffraction, X-ray photoelectron spectroscopy, Ultraviolet (UV) photoelectron spectrometer, UV–vis spectroscopy, Transmission Electron Microscope (TEM), and Raman spectroscopy were used to characterize the properties of Zn–ZrO_2_ NPs and explore the mechanisms behind the SERS phenomenon. The charge transfer (CT) process is considered to be responsible for the SERS performance of 4–MBA adsorbed on Zn–ZrO_2_. The results of this study demonstrate that an appropriate doping ratio of Zn ions can promote the charge transfer process between ZrO_2_ NPs and probe molecules and significantly improve the SERS properties of ZrO_2_ substrates.

## 1. Introduction

Since it was first discovered in 1974, surface-enhanced Raman scattering (SERS) has been considered to be an effective and promising spectroscopic technique [1]. Due to its high sensitivity, high selectivity, rapidity, high resolution, and nondestructive examination, it has rapidly gained ground in various fields from analytical chemistry to medical science [2,3,4,5]. Recently, semiconductors have, as a new type SERS active material, been widely investigated. Compared with the traditional metal substrates, semiconductor substrates have many impressive advantages, including their high stability, low cost, excellent controllability, and environment protection [6,7,8,9,10]. Since a better understanding of the mechanisms of the SERS phenomenon will benefit investigating SERS substrates, researchers are committed to exploring the mechanisms behind the enhancement of the SERS effect. The most likely explanations are electromagnetic mechanisms (EM), as well as chemical mechanisms (CM) due to the charge transfer (CT) process between the probe molecules and substrate [11,12,13,14,15,16]. For semiconductor substrates, the primary mechanism of the SERS phenomenon is likely the CT process. However, the number of semiconductor substrates is still limited, and the enhancement mechanism of SERS still needs further investigation.

Zirconium dioxide (ZrO_2_) is an n-type semiconductor with a large number of oxygen vacancy defects on its surface. Recently, it was found that ZrO_2_ exhibited a luminescence effect, which is caused by a transition between the new state of trapped electron oxygen vacancies and the ground state energy level [17]. Therefore, ZrO_2_ has become a remarkable material to promote the CT process and SERS phenomenon. Additionally, the application of nanosized ZrO_2_ has become an area of focus in the research of nanoceramic technology [18]. It has broad applications and developments in industrial synthesis, catalyst carriers, catalysts, medical materials and refractory materials [19,20,21,22]. This research has broadened the application of SERS substrates and is helpful for a better characterization of the basic properties of ZrO_2_. As of now, we still know little about ZrO_2_ as a SERS substrate, and thus, further studies to investigate higher SERS properties are essential. Therefore, it is vital to increase the enrichment of the surface defects of ZrO_2_ nanoparticles (NPs). It is well known that doping with metal ions can improve the optical and catalytic properties of semiconductor NPs, thus leading to third generation photoactive materials [23]. In recent years, the metal ion-doped semiconductor system has been employed to research the CT mechanism of the SERS phenomenon. As reported before, some metal ions, such as Zn, Mn, and Co, can be utilized as the dopant in the semiconductor to improve the surface properties. For example, the Zn-doped TiO_2_ NPs, Co-doped ZnO NPs and Mn-doped TiO_2_ NPs have been studied to provide a better SERS enhancement than a pure semiconductor substrate [24,25,26]. Thus, the metal Zn dopant is considered in this study.

In this study, we fabricated ZrO_2_ and Zn–ZrO_2_ NPs, with Zn doping concentrations from 0.5 to 5%, by the sol-gel method, as active SERS substrates. The results have shown an evident enhancement caused by the Zn dopant compared to the pure ZrO_2_. It is noteworthy that ZrO_2_ NPs with a 1% Zn doping concentration show the highest SERS enhancement. To further evaluate the enhancement ability, we also calculated the enhancement factor of the probe molecule (4–MBA) adsorbed on (1%) Zn–ZrO_2_ NPs, which is up to 10^4^. A possible mechanism between Zn–ZrO_2_ NPs and the 4–MBA molecule is also described in this paper. On one hand, this study has value in improving the SERS properties and broadening the applications of ZrO_2_ NPs as SERS substrates. On the other hand, it is helpful for understanding of the mechanism of the SERS phenomenon on the semiconductor substrate. As a nontoxic, and highly biocompatible SERS substrate, the applications of ZrO_2_ nanophase materials have bright prospects in catalysis, biomedical, chemical analysis and other industries [27,28].

## 2. Materials and Methods

### 2.1. Chemical Reagents

Zirconium nitrate pentahydrate (Zr(NO_3_)_4_·5H_2_O) and zinc nitrate trihydrate (Zn(NO_3_)_2_·3H_2_O) were purchased from the State Group Chemical Reagent Co., Ltd. (Shanghai, China ) Triethylamine (TEA) and hexadecyl trimethyl ammonium bromide (CTAB) were purchased from the Beijing Dingguo Biotech Co., Ltd (Beijing, China). 4–Mercaptobenzoic acid (4–MBA) was purchased from Sigma-Aldrich (Shanghai, China). All reagents used are of analytical grade with no further purification.

### 2.2. Synthesis of ZrO_2_ and Zn–ZrO_2_ NPs

ZrO_2_ and Zn–ZrO_2_ NPs were synthesized by the sol-gel process [29]. First, 8.72 g of Zr(NO_3_)_4_·5H_2_O and 1.475 g of hexadecyl trimethyl ammonium bromide were dissolved in 90 mL of deionized water at room temperature. A series of appropriate amounts of Zn(NO_3_)_2_·3H_2_O (the molar ratios of Zn to Zr were 0.5%, 1%, 3% and 5%) were added in the mixed solution. After complete dissolution, 11.308 mL of triethylamine solution was added. Then, the mixture was placed at 75 °C under continuous stirring for 8 h. Next, the product was separated by centrifugation and dried at 82 °C for 48 h, followed by elution with ethanol for 20 h. Finally, ZrO_2_ and Zn–ZrO_2_ NPs with different Zn doping concentrations were obtained by calcination in a muffle furnace for 2 h at 500 °C.

### 2.3. Adsorption of Probe Molecules

**Figure 1:**
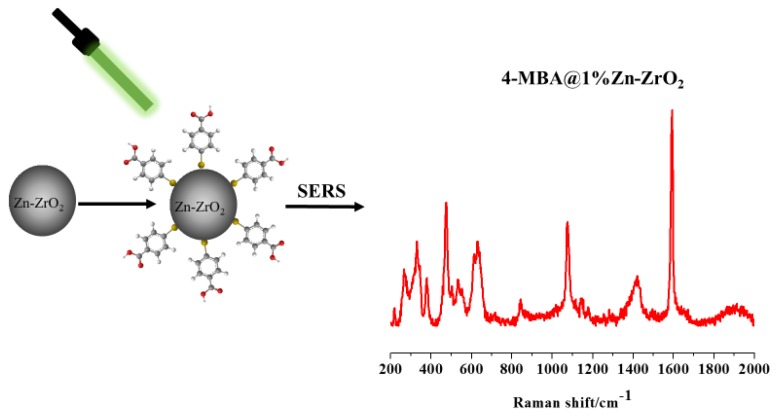
Diagram of the Zn–ZrO_2_ (1%) NPs and 4–MBA.

The modified ZrO_2_ and Zn–ZrO_2_ NPs were obtained by the following experimental methods: 10 mL of a 4–MBA ethanol solution with a molar concentration of 1 × 10^−3^ M was extracted, and 20 mg of ZrO_2_ as well as four groups of Zn–ZrO_2_ NPs with different doping ratios were uniformly dispersed in 4–MBA ethanol solution. The turbid liquid was stirred at room temperature for 4 h. Then, the precipitates were separated by centrifugation, and the precipitates were washed repeatedly by deionized water and ethanol solution to remove the probe molecules that were not adsorbed on the NPs. Finally, the 4–MBA-modified ZrO_2_ and Zn–ZrO_2_ NPs were respectively kept in glass slides for the subsequent Raman measurement (Figure 1). 

### 2.4. Characterization of Materials

The crystal structures of the ZrO_2_ and Zn–ZrO_2_ samples were determined by X-ray diffraction (XRD) using a Rikagu Smartlab X-ray powder diffractometer (Tokyo, Japan) with 0.15406 nm radiation. The morphologies of the ZrO_2_ and Zn–ZrO_2_ NPs were characterized by using a JEM-2000EX TEM instrument (Tokyo, Japan). The surface electronic valence state for the UV photoelectron spectrometer (UPS) as well as the elemental composition of the samples were investigated by X-ray photoelectron spectroscopy (XPS) with a Thermofisher Escalab 250XI (Waltham, MA, USA). The UV-vis diffuse reflectance spectroscopy (DRS) spectra were recorded on a Cary 5000 UV-vis spectrophotometer (Santa Clara, CA, USA). The Raman spectra were studied with a Horiba HR Evolution Raman spectrometer (Paris, France). The 532 nm laser irradiation from a 20 mW air-cooled argon ion laser was used as an exciting source. The laser was focused on the white point of ZrO_2_ and Zn–ZrO_2_ NPs under Halogen Lamp irradiation. The microscope attachment was based on a Leica DMLM system, and a 50× long range objective was used to focus the laser beam onto a spot 1 µm in diameter. The typical exposure time for each sample in this study was 10 s, with three accumulations at room temperature. There are three batches of test samples, and the Raman intensities were calculated based on the three independent measurements.

## 3. Results and Discussion

### 3.1. Properties of ZrO_2_ and Zn–ZrO_2_

#### 3.1.1. Characterization by XRD and TEM of the ZrO_2_ and Zn–ZrO_2_ NPs

**Figure 2:**
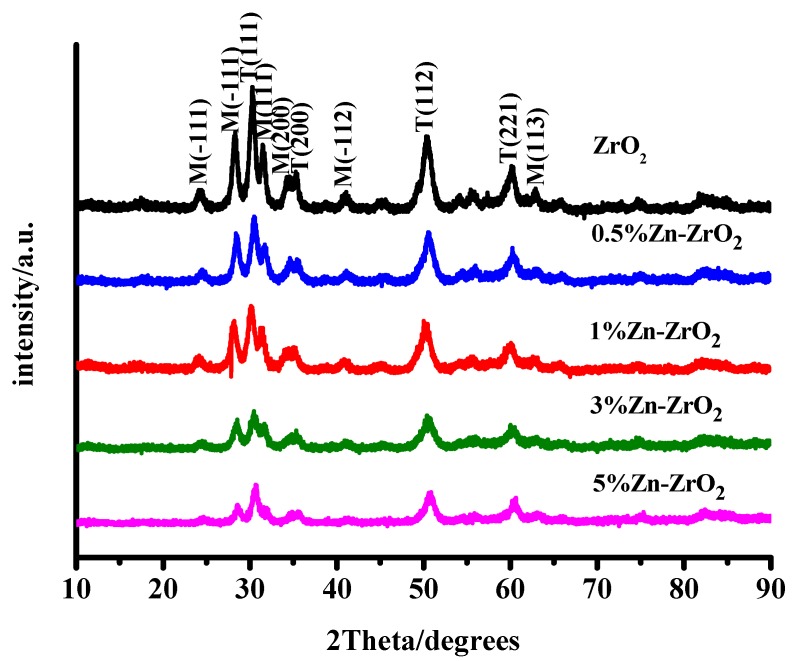
XRD diagrams of the ZrO_2_ and Zn–ZrO_2_ (0.5–5%) NP samples.

**Figure 3:**
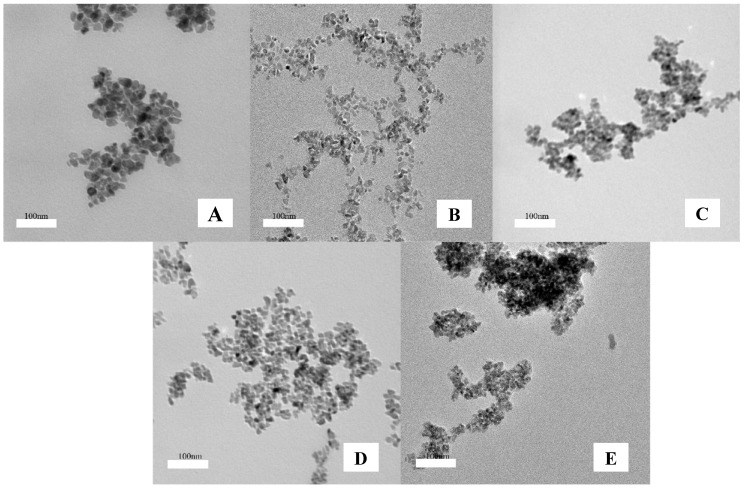
TEM images of the (**A**) ZrO_2_ and (**B**,**C**,**D**, and **E**: 0.5, 1, 3, and 5%) Zn–ZrO_2_ NPs. The scale bar represents 100 nm.

Zirconia is a remarkable material, which includes three polymorphs: cubic, tetragonal and monoclinic. The XRD spectra of the ZrO_2_ and Zn–ZrO_2_ (0.5–5%) NPs are shown in Figure 2. It presents that at a temperature of 500 °C most of the reflection peaks indicated the formation of a monoclinic phase (m-ZrO_2_) (PDFcard: 13–307) and a small portion of a tetragonal phase (t-ZrO_2_) (PDF card: 88-1007). Among the five samples (from 0% to 5%), there were no other XRD diffraction peaks, with the exception of the ZrO_2_ crystalline phase, which indicates that the doped Zn ions disperse among the ZrO_2_ crystallites rather than exist in the form of ZnO_2_. Furthermore, as the half width of the peak at 2θ = 30.2° relates to the degree of crystallinity, it can be summarized from Figure 2 that the size of the crystallite decreases with increasing Zn concentrations. This is probably because the doped Zn ions slightly inhibit the growth of the ZrO_2_ lattice. The crystal diameters were approximately 10.5, 11, 10.8, 10.2 and 8.6 nm, respectively, calculated by the Scherrer formula: D = kλ/(β cos θ) [30]. Figure 3 presents the TEM images of the ZrO_2_ and Zn–ZrO_2_ (0.5–5%) NPs. The results show that the ZrO_2_ and Zn-ZrO_2_ particles have different sizes, and are also irregular and sphere-like, which agrees well with the XRD results.

#### 3.1.2. Raman Spectra of ZrO_2_ and Zn–ZrO_2_ NPs

**Figure 4:**
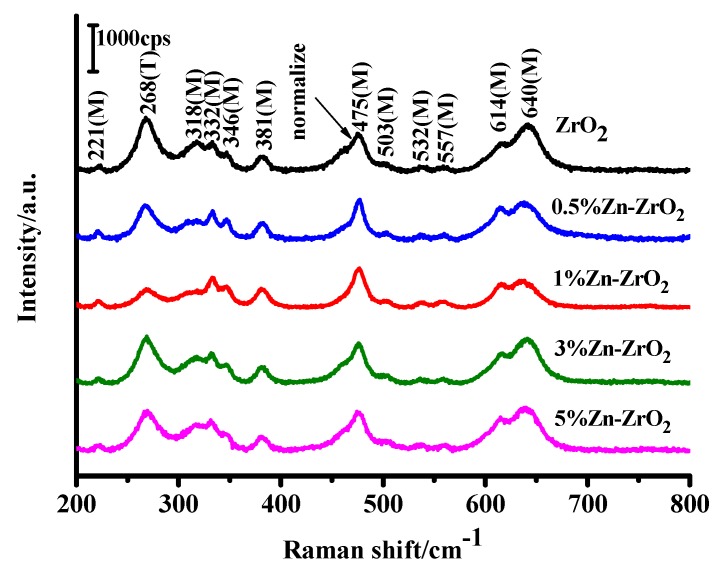
Raman spectra of the ZrO_2_ and Zn–ZrO_2_ (0.5–5%) samples.

The Raman spectra of ZrO_2_ and Zn–ZrO_2_ NPs in the range of 200 to 800 cm^−1^ are shown in Figure 4. The crystal structures of the ZrO_2_ and Zn–ZrO_2_ NPs can be determined by the Raman shift (Raman vibration frequency). We can clearly observe from the Raman measurement that all of the Zn–ZrO_2_ samples mostly exist in the m-phase, which is in agreement with the XRD results [31]. Interestingly, with the increase of the Zn doping, the peaks located at 268 and 318 cm^−1^ were constantly decreased. When the doping content is 1%, the change is the most significant, which is considered to be the result of increasing surface defects, such as oxygen vacancies. This is consistent with the results observed in the XRD spectra [32].

#### 3.1.3. XPS Measurements

**Figure 5:**
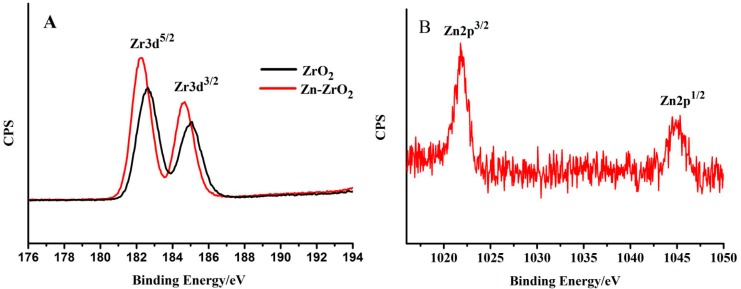
The XPS spectra of (**A**) Zr 3d and (**B**) Zn 2p in the ZrO_2_ and Zn–ZrO_2_ (1%) NPs.

**Figure 6:**
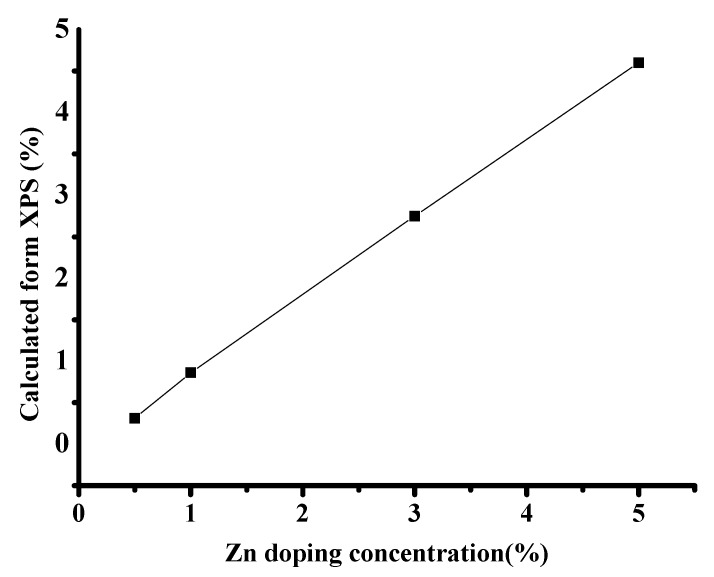
The surface concentration of Zn ions on the Zn–ZrO_2_ NPs.

XPS was employed to estimate the elemental composition, as well as the chemical and electronic states, of the ZrO_2_ and (1%) Zn–ZrO_2_ samples. The results (Figure 5A and Figure S1) show that, compared with the pure ZrO_2_ NPs, the chemical state of Zr in the Zn–ZrO_2_ system remains unchanged but induces the O1s binding energy to increase from 530 to 530.4 eV. It can also be observed that the position of the Zr 2p peak shifts toward a lower direction compared to the bare ZrO_2_, which indicates that Zn ions do have an effect on the ZrO_2_ lattice [33,34]. For Zn, in the XPS spectra of Zn 2p (Figure 5B), we observe two obvious peaks at 1021.8 and 1044.9 eV, which indicate the Zn 2p_3/2_ and Zn 2p_1/2_. This verifies that Zn disperses among the ZrO_2_ lattice and exists in the form of divalent ions, which supports the conclusion of the XRD results. In addition, the surface coverages of Zn ions on Zn–ZrO_2_ evaluated from the XPS results are 0%, 0.31%, 0.86%, 2.8%, and 4.6%, respectively (Figure 6). This proves that the Zn was doped in ZrO_2_, although the results are slightly lower than the Zn content that we used.

### 3.2. Measurement of UV-Vis DRS

**Figure 7:**
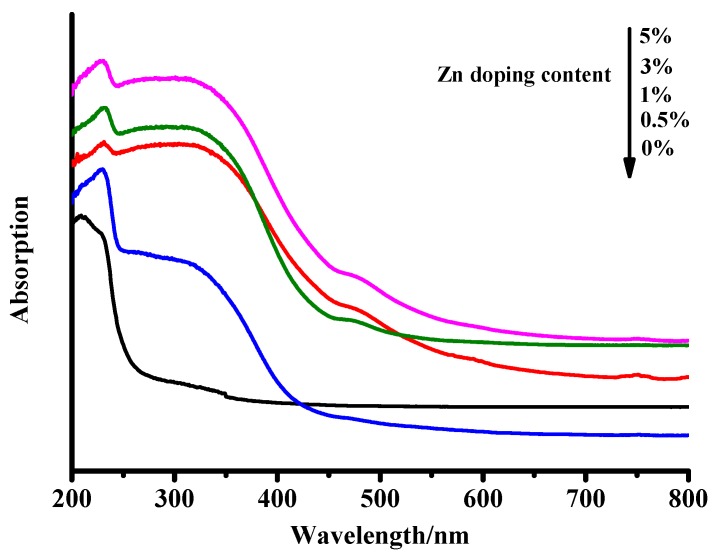
The UV-vis DRS spectra of the ZrO_2_ and Zn–ZrO_2_ NPs.

UV-vis spectroscopy was applied to further analyze the optical properties of the ZrO_2_ and Zn–ZrO_2_ NPs. Figure 7 exhibits the UV-vis DRS spectra of ZrO_2_ with different Zn doping contents. The wide optical absorption below 250 nm for the five samples is attributable to the band-band electron transition of ZrO_2_. We observe that the absorption curves are redshifted, and the shift becomes more obvious with increasing Zn doping concentrations, which is because of the increasing defect concentration with the increasing amount of Zn ions [35]. In addition, according to the Kubelka-Munk formula, the band gaps of the ZrO_2_ and Zn–ZrO_2_ NPs can be calculated to be 4.95, 3.03, 2.86, 2.89, and 3.08 eV, respectively, as shown in Figure S2. The band gap generally refers to the distance between the top of the valence band (VB) and the bottom of the conduction band (CB) [36]. For semiconductors, the band gap reflects the possibility that the electrons are excited. The narrower the band gap is, the easier it will be to excite the electrons.

### 3.3. SERS Spectra of 4-MBA Adsorbed on ZrO_2_ and Zn–ZrO_2_ NPs

**Figure 8:**
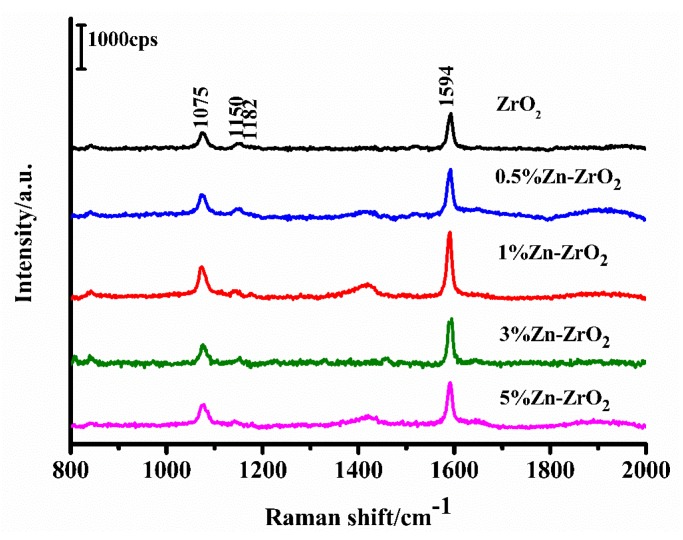
The SERS spectra of 4–MBA adsorbed on the ZrO_2_ and Zn–ZrO_2_ NPs.

The SERS spectra of the 4–MBA molecules absorbed on the ZrO_2_ and Zn–ZrO_2_ NPs with different Zn doping contents (0%, 0.5%, 1%, 3%, and 5%) are shown in Figure 8. Obvious 4–MBA Raman signal peaks were observed on the SERS spectra at 1075, 1150, 1182, and 1594 cm^−1^. All of these peaks are in agreement with the reported literature [7,30,37]. The strong bands located at 1075 and 1594 cm^−1^ are assigned to the aromatic ring vibration with a C-S stretching mode (ν12a, a1) as well as the aromatic ring characteristic vibrations (ν8a, a1), respectively. The weak bands at 1150 (ν15, b2) and 1182 cm^−1^ (ν9, a1) correspond to the C–H deformation modes. It is worth noting that the b2 mode is mainly attributed to the Herzberg-Teller contribution, which induces enhanced CT effects. The intensities of the SERS signals first increased (from pure ZrO_2_ to 1% Zn–ZrO_2_) and then decreased (from 1% Zn–ZrO_2_ to 5% Zn–ZrO_2_). It can be clearly seen from Figure 8 that the 1% Zn doping concentration has the best SERS enhancement effect.

In addition, it is notable that the SERS signal of the Zn ion-doped substrate is higher than the signal of the undoped substrate, and that the optimum Zn ion concentration is 1%. The reasons behind these phenomena are considered to be as follows. The doped Zn ions tend to improve the enrichment of the surface defect state (oxygen vacancies) of ZrO_2_ and enhance its SERS signal. When the concentration of Zn ions further increases, the increased surface defects promote the electrons to bind to oxygen vacancies, which suppresses the CT process between the substrate and the adsorbed molecules, thus weakening the SERS signal.

### 3.4. Enhancement Factor (EF) of Zn–ZrO_2_ NPs

**Figure 9:**
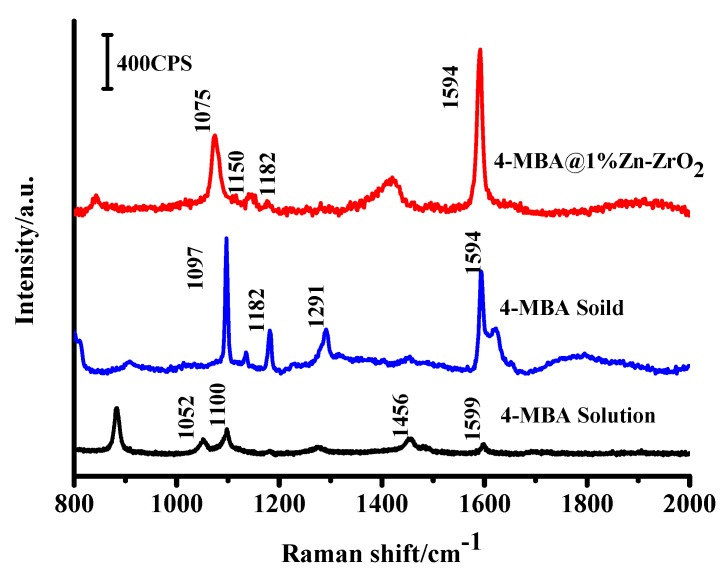
**The** SERS spectra of 4–MBA adsorbed on the Zn–ZrO_2_ (1%) NPs, the 4–MBA bulk sample and ethanol solution (1 × 10^−1^ M).

To carry out an evaluation of the enhancement ability, we calculated the EF of the 4–MBA adsorbed on the Zn–ZrO_2_ (1%) NPs under a 532 nm laser illumination. Here, we used the ν(C–C) aromatic ring characteristic vibrations (~1594 cm^−1^) to calculate the surface enhancement factor. We calculated the EF according to the following equation [38]:

EF = I_Surf_N_Bulk_/I_Bulk_N_Surf_ (1)

where N and I represent the number of the adsorbed and pure 4–MBA molecules and SERS intensities of the adsorbed 4–MBA on the Zn–ZrO_2_ NPs and the bulk powder of 4–MBA. I_surf_ is the intensity of the SERS band at 1594 cm^−1^, and I_Bulk_ is the intensity of the Raman band at 1594 cm^−1^ of the powder sample. N_Surf_ relates to the number of 4–MBA absorbed on the Zn–ZrO_2_ NPs, and N_Bulk_ is the number of 4–MBA powder under laser illumination. Based on the volume of 4–MBA and its density (1.5 g·cm^−3^), we calculated that N_Bulk_ = 7.61 × 10^10^. Based on the results of the XRD spectra, the average diameter of the 1% Zn–ZrO_2_ samples can be calculated to be 10.8 nm by the Scherrer formula. Assuming a monolayer of 4–MBA coverage on the Zn–ZrO_2_ surface, we estimated N_Surf_ ≈ 6.15 × 10^6^ by using the probe spot size of the laser and the boundary density of the adsorbed 4–MBA. Figure 9 shows that the intensity ratio of I_Surf_ to I_Bulk_ is 1.57. Therefore, the surface enhancement factor (EF) can be calculated as 1.94 × 10^4^ according to Equation (1).

The result shows that the EF value of 4–MBA adsorbed on the Zn–ZrO_2_ NPs is greater than 10^4^. By using the same calculation method, we calculated the surface enhancement factor of the ZrO_2_ substrate as 4.32 × 10^3^. The EF values of 4–MBA adsorbed on the pure ZrO_2_ and Zn–ZrO_2_ NPs are listed in Table S1. Based on these calculations, we find that, compared to the pure ZrO_2_ NPs, the doped Zn ions remarkably enhance the SERS properties of the ZrO_2_ substrate, which increased by an order of magnitude.

### 3.5. CT Mechanism and Direction of CT Effects

The mechanism of the SERS effect has been a research hotspot recently. By far, one generally accepted mechanism is the electromagnetic mechanism (EM). The EM relates to the mutual effect between the local electric field increase, which is excited by the incident laser, and the local electric field generated by probe molecules. Another mechanism is called the chemical mechanism (CM). When the substrate is a semiconductor material, there is an interaction of chemical adsorption and photo-induced transfer between the semiconductor and probe molecules. Based on the related literature, the surface plasmon resonance effect of semiconductor nanomaterials is located in the near-infrared or infrared region. Herein, the excitation line used in this experiment is 532 nm; therefore, the CT process has become a very real possibility [39]. Additionally, in the SERS spectrum, we can observe a not totally symmetric C–H deformation mode (ν15, b2) at 1150 cm^−1^, which can be only explained by the CT effect. For the abovementioned reasons, we consider that the CT mechanism is responsible for the observed SERS phenomena in the Zn–ZrO_2_-4–MBA system.

**Figure 10:**
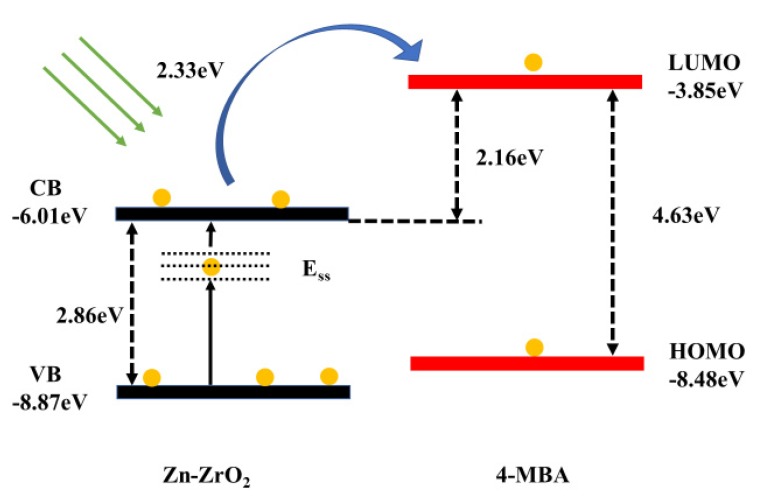
Diagram of the CT mechanism of 4–MBA adsorbed on the 1% Zn-doped ZrO_2_ NPs.

Since the CT mechanism is a kind of resonance Raman process, in the Zn–ZrO_2_-4–MBA system, the process of charge transfer occurs between the Zn–ZrO_2_ substrate and the 4–MBA probe molecules. The energy levels of the lowest unoccupied molecular orbital (LUMO) and the highest occupied molecular orbital (HOMO) of the 4–MBA molecules, which were −3.85 and −8.48 eV, respectively, referred to [26], as presented in Figure 10. The energy levels of Zn–ZrO_2_ were determined by UPS spectra (Figure S3). By our calculation, the valence band (VB) of Zn–ZrO_2_ is situated at −8.87 eV [40]. According to the calculation results from the UV-vis DRS spectra, the band gap between the VB and CB is 2.86 eV, and thus the conduction band (CB) of Zn–ZrO_2_ can be located at −6.01 eV. Since the band gap (2.86 eV) from the VB to the CB is larger than the energy (2.33 eV) that the incident light contains, it is impossible to excite electrons between them. It can be observed that the energy is not enough for the excitation from the LUMO level (−3.85 eV) to the HOMO level (−8.48 eV) either. However, the ZrO_2_ NPs are rich in surfaces defects, such as oxygen vacancy defects. Based on the XPS results and UV-vis spectra discussed above, the doped Zn ions further increase the surface defect content. These surface defects tend to catch electrons and form the surface states energy level (E_SS_), which is supposed to be located between the VB and CB of the Zn–ZrO_2_ NPs [41].

Therefore, a reasonable CT model between the Zn–ZrO_2_ NPs and the 4–MBA molecule is illustrated in Figure 10. The whole charge transfer process may be divided into three steps. With a laser of 532 nm (2.33 eV), the incident light first excites the electrons from the VB of the Zn–ZrO_2_ to the ESS. Then, these electrons were injected into the CB of the Zn–ZrO_2_ with the aid of the excitation of the laser. Finally, the excited electrons, which were derived from the CB of the Zn–ZrO_2_, transition to the LUMO level of the adsorbed 4–MBA molecules.

The doped metal ions can help to enrich the surface state of the NPs and then improve the surface properties of the semiconductor NPs. Surface defects make a great contribution to the Zn–ZrO_2_-to-4–MBA CT process. Surface defects can raise the photocarrier separation efficiency as a photoelectron capture trap and then form a necessary intermediate state, as shown in Figure 10. Therefore, we employed Zn-doped ZrO_2_ NPs as a type of new SERS-active substrate and demonstrated the validity and universality of the CT mechanism in the semiconductor SERS phenomenon.

## 4. Conclusions

In this study, we fabricated ZrO_2_ and Zn–ZrO_2_ NPs with different Zn-doping concentrations as SERS substrates with the sol-gel method. The findings of this study show that the doped Zn ions significantly improve the SERS properties of ZrO_2_ NPs, and that the 1% Zn doping concentration NPs exhibit the highest SERS enhancement on the surface-adsorbed probe molecule. The enhancement can also be proven by the calculation results of the enhancement factor (EF), which is an order of magnitude higher than that of the pure ZrO_2_. Moreover, the CT mechanism is considered to be responsible for the SERS performances of adsorbed 4–MBA on the Zn–ZrO_2_ substrate. It is expected that this study will not only be valuable in improving the SERS properties and broadening the applications of ZrO_2_ NPs, but that it will also provide a more in-depth understanding of the mechanism of the SERS phenomenon on semiconductor substrates.

## Supplementary Materials

The following are available online at https://www.mdpi.com/2079-4991/9/7/983/s1, Figure S1: XPS spectra of O1s in ZrO_2_ and Zn-ZrO_2_ (1%) NPs, Figure S2: UV-vis DRS spectra of (A) pure ZrO_2_ nanoparticle, and Zn-doped ZrO_2_ nanoparticle (B) 0.5% (C) 1% (D) 3% (E) 5%, Figure S3: The ultraviolet photoelectron spectroscopy (UPS) of Zn-ZrO_2_ (1%) nanoparticles, Table S1: Enhancement factor of 4-MBA adsorbed on ZrO_2_ and Zn-ZrO_2_ NPs (0.5%, 1%, 3%, and 5%).

## References

[1] Fleischmann M., Hendra P.J., McQuillan A.J. (1974). Raman spectra of pyridine adsorbed at a silver electrode. Chem. Phys. Lett..

[2] Muehlethaler C., Leona M., Lombardi J.R. (2015). Review of surface enhanced Raman scattering applications in forensic science. Anal. Chem..

[3] Liang D., Jin Q., Yan N., Feng J., Wang J., Tang X. (2018). SERS nanoprobes in biologically Raman silent region for tumor cell imaging and in vivo tumor spectral detection in mice. Adv. Biosyst..

[4] Mao Z., Liu Z., Chen L., Yang J., Zhao B., Jung Y.M., Wang X., Zhao C. (2013). Predictive value of the surface-enhanced resonance Raman scattering-based mtt assay: A rapid and ultrasensitive method for cell viability in situ. Anal. Chem..

[5] Chen L., Yu Z., Lee Y., Wang X., Zhao B., Jung Y.M. (2012). Quantitative evaluation of proteins with bicinchoninic acid (bca): Resonance Raman and surface-enhanced resonance Raman scattering-based methods. Analyst.

[6] Han X.X., Ji W., Zhao B., Ozaki Y. (2017). Semiconductor-enhanced Raman scattering: Active nanomaterials and applications. Nanoscale.

[7] Sun Z., Zhao B., Lombardi J.R. (2007). ZnO nanoparticle size-dependent excitation of surface Raman signal from adsorbed molecules: Observation of a charge-transfer resonance. Appl. Phys. Lett..

[8] Otto A. (2005). The ‘chemical’ (electronic) contribution to surface-enhanced Raman scattering. J. Raman Spectrosc..

[9] Islam S.K., Tamargo M., Moug R., Lombardi J.R. (2013). Surface-enhanced Raman scattering on a chemically etched ZnSe surface. J. Phys. Chem. C.

[10] Wu H., Wang H., Li G. (2017). Metal oxide semiconductor sers-active substrates by defect engineering. Analyst.

[11] Huang Y.F., Zhang M., Zhao L.B., Feng J.M., Wu D.Y., Ren B., Tian Z.Q. (2014). Activation of oxygen on gold and silver nanoparticles assisted by surface plasmon resonances. Angew. Chem. Int. Ed..

[12] Hu J.-W., Zhang Y., Li J.-F., Liu Z., Ren B., Sun S.-G., Tian Z.-Q., Lian T. (2005). Synthesis of Au@Pd core–shell nanoparticles with controllable size and their application in surface-enhanced Raman spectroscopy. Chem. Phys. Lett..

[13] Mogensen K.B., Kneipp K. (2014). Size-dependent shifts of plasmon resonance in silver nanoparticle films using controlled dissolution: Monitoring the onset of surface screening effects. J. Phys. Chem. C.

[14] Jiang X., Sun X., Yin D., Li X., Yang M., Han X., Yang L., Zhao B. (2017). Recyclable au-TiO_2_ nanocomposite sers-active substrates contributed by synergistic charge-transfer effect. Phys. Chem. Chem. Phys. PCCP.

[15] Lombardi J.R., Birke R.L. (2014). Theory of surface-enhanced Raman scattering in semiconductors. J. Phys. Chem. C.

[16] Liu L., Yang H., Ren X., Tang J., Li Y., Zhang X., Cheng Z. (2015). Au–ZnO hybrid nanoparticles exhibiting strong charge-transfer-induced sers for recyclable sers-active substrates. Nanoscale.

[17] Navio J., Hidalgo M., Colon G., Botta S., Litter M. (2001). Preparation and physicochemical properties of ZrO_2_ and Fe/ZrO_2_ prepared by a sol−gel technique. Langmuir.

[18] Li X., Xu Y., Mao X., Zhu Q., Xie J., Feng M., Jiang B., Zhang L. (2018). Investigation of optical, mechanical, and thermal properties of ZrO_2_-doped Y_2_O_3_ transparent ceramics fabricated by HIP. Ceram. Int..

[19] Duan H., Unno M., Yamada Y., Sato S. (2017). Adsorptive interaction between 1,5-pentanediol and mgo-modified ZrO_2_ catalyst in the vapor-phase dehydration to produce 4-penten-1-ol. Appl. Catal. A Gen..

[20] Kaviyarasu K., Kotsedi L., Simo A., Fuku X., Mola G.T., Kennedy J., Maaza M. (2017). Photocatalytic activity of ZrO_2_ doped lead dioxide nanocomposites: Investigation of structural and optical microscopy of RhB organic dye. Appl. Surf. Sci..

[21] Marti A. (2000). Inert bioceramics (Al_2_O_3_, ZrO_2_) for medical application. Injury.

[22] Zhou H., Shen Y., Xi J., Qiu X., Chen L. (2016). ZrO_2_-nanoparticle-modified graphite felt: Bifunctional effects on vanadium flow batteries. ACS Appl. Mater. Interfaces.

[23] Gionco C., Paganini M.C., Giamello E., Burgess R., Di Valentin C., Pacchioni G. (2014). Cerium-doped zirconium dioxide, a visible-light-sensitive photoactive material of third generation. J. Phys. Chem. Lett..

[24] Yang L., Zhang Y., Ruan W., Zhao B., Xu W., Lombardi J.R. (2009). Improved surface-enhanced Raman scattering properties of TiO_2_ nanoparticles by Zn dopant. J. Raman Spectrosc..

[25] Xue X., Ruan W., Yang L., Ji W., Xie Y., Chen L., Song W., Zhao B., Lombardi J.R. (2012). Surface-enhanced Raman scattering of molecules adsorbed on co-doped ZnO nanoparticles. J. Raman Spectrosc..

[26] Xue X., Ji W., Mao Z., Li Z., Ruan W., Zhao B., Lombardi J.R. (2012). Effects of Mn doping on surface enhanced Raman scattering properties of TiO_2_ nanoparticles. Spectrochim. Acta. Part AMol. Biomol. Spectrosc..

[27] Long D., Niu M., Tan L., Fu C., Ren X., Xu K., Zhong H., Wang J., Li L., Meng X. (2017). Ball-in-ball ZrO_2_ nanostructure for simultaneous CT imaging and highly efficient synergic microwave ablation and tri-stimuli responsive chemotherapy of tumor. Nanoscale.

[28] Lu Z., Zhu Z., Zheng X., Qiao Y., Guo J., Li C.M. (2011). Biocompatible fluorescence-enhanced ZrO_2_-CdTe quantum dot nanocomposite for in vitro cell imaging. Nanotechnology.

[29] Zhou L., Xu J., Li X., Wang F. (2006). Metal oxide nanoparticles from inorganic sources via a simple and general method. Mater. Chem. Phys..

[30] Yang L., Jiang X., Ruan W., Zhao B., Xu W., Lombardi J.R. (2008). Observation of enhanced Raman scattering for molecules adsorbed on TiO_2_ nanoparticles: Charge-transfer contribution. J. Phys. Chem. C.

[31] Carlone C. (1992). Raman spectrum of zirconia-hafnia mixed crystals. Phys. Rev. B.

[32] Zhao X., Vanderbilt D. (2002). Phonons and lattice dielectric properties of zirconia. Phys. Rev. B.

[33] Palma-Goyes R.E., Vazquez-Arenas J., Ostos C., Manzo-Robledo A., Romero-Ibarra I., Calderón J.A., González I. (2018). In search of the active chlorine species on Ti/ZrO_2_-RuO_2_-Sb_2_O_3_ anodes using dems and xps. Electrochim. Acta.

[34] Guittet M.J., Crocombette J.P., Gautier-Soyer M. (2001). Bonding and xps chemical shifts in ZrSiO_4_ versus SiO_2_ and ZrO_2_: Charge transfer and electrostatic effects. Phys. Rev. B.

[35] Xue X., Ji W., Mao Z., Zhao C., Zhao B., Lombardi J.R. (2013). Simultaneous enhancement of phonons modes with molecular vibrations due to mg doping of a TiO_2_ substrate. RSC Adv..

[36] Chang S.-m., Doong R.-a. (2007). Interband transitions in sol−gel-derived ZrO_2_ films under different calcination conditions. Chem. Mater..

[37] Jiang L., Yin P., You T., Wang H., Lang X., Guo L., Yang S. (2012). Highly reproducible surface-enhanced Raman spectra on semiconductor SnO_2_ octahedral nanoparticles. ChemPhysChem.

[38] Jiang L., You T., Yin P., Shang Y., Zhang D., Guo L., Yang S. (2013). Surface-enhanced Raman scattering spectra of adsorbates on Cu_2_O nanospheres: Charge-transfer and electromagnetic enhancement. Nanoscale.

[39] Wang Y., Ruan W., Zhang J., Yang B., Xu W., Zhao B., Lombardi J.R. (2009). Direct observation of surface-enhanced Raman scattering in zno nanocrystals. J. Raman Spectrosc..

[40] Zhang X., Yu Z., Ji W., Sui H., Cong Q., Wang X., Zhao B. (2015). Charge-transfer effect on surface-enhanced Raman scattering (SERS) in an ordered Ag NPs/4-mercaptobenzoic acid/TiO_2_ system. J. Phys. Chem. C.

[41] Yang L., Qin X., Gong M., Jiang X., Yang M., Li X., Li G. (2014). Improving surface-enhanced Raman scattering properties of TiO_2_ nanoparticles by metal co doping. Spectrochim. Acta. Part AMol. Biomol. Spectrosc..

