# Supplementary material for: Improved Surface-Enhanced Raman Scattering Properties of ZrO2 Nanoparticles by Zn Doping
Source: Nanomaterials (Basel). 2019 Jul 6;9(7):983. doi: 10.3390/nano9070983 (PMC6669659; doi:10.3390/nano9070983)
Supplement: Supplementary file 1 [file nanomaterials-09-00983-s001.pdf]

Supplementary Material

Improved Surface-enhanced Raman Scattering Properties of ZrO_2 Nanoparticles by Zn Doping

Peng Ji[1,2], Zhu Mao[2], Zhe Wang[1], Xiangxin Xue[3] , Yu Zhang[2], Jiaao Lv[1] and Xiumin Shi[1,*]

[1] College of Chemical Engineering, Changchun University of Technology, Changchun, China
[2] School of Chemistry and Life Science, Changchun University of Technology, Changchun, China
[3] Key Laboratory of Preparation and Applications of Environmental Friendly Materials, Jilin Normal University, Ministry of Education, Changchun, China
* Correspondence: shixiumin@ccut.edu.cn; Tel.: +86-431-8571-6463

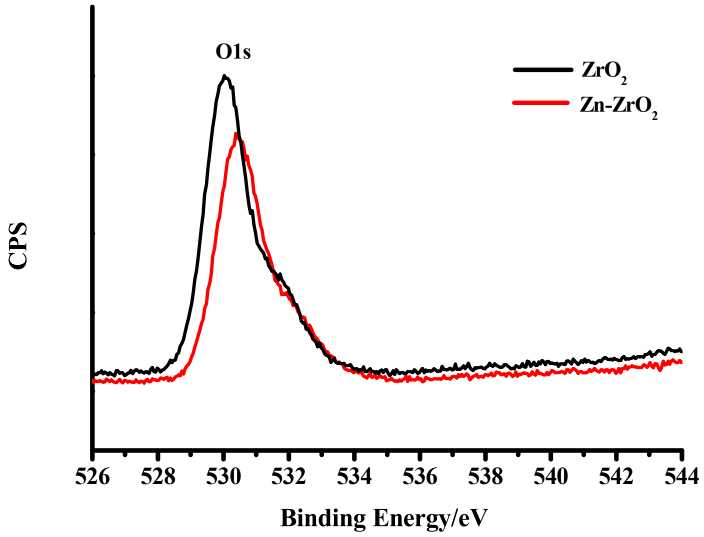

Figure S1. XPS spectra of O1s in ZrO_2 and Zn-ZrO_2 (1%) NPs.

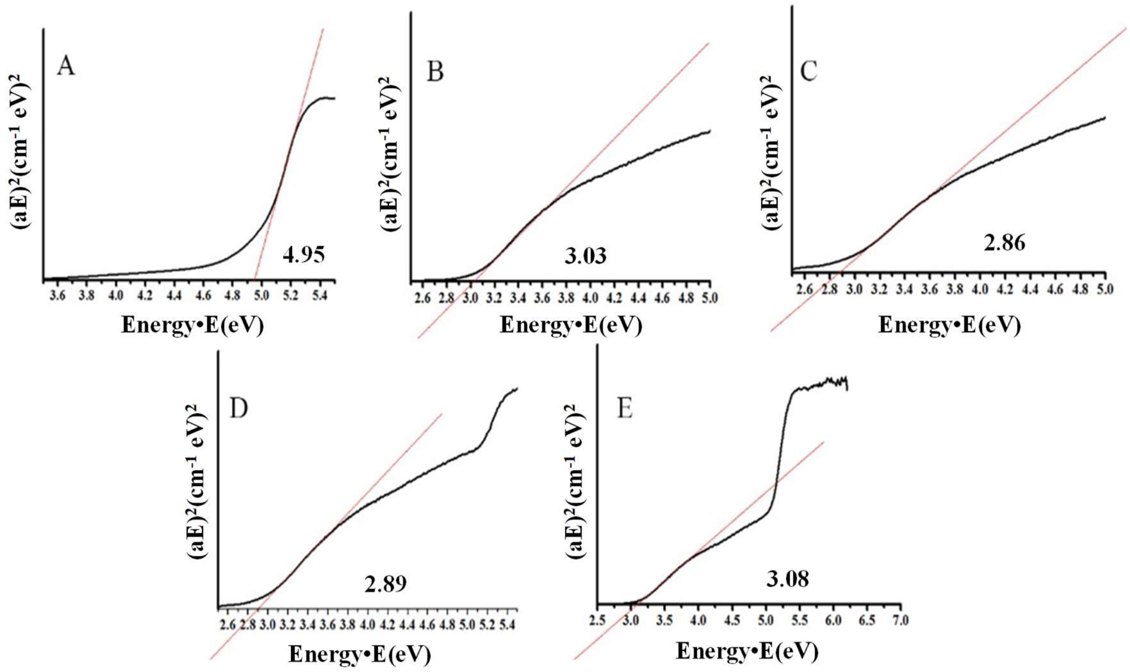

Figure S2. UV-vis DRS spectra of (A) pure ZrO_2 nanoparticle, and Zn-doped ZrO_2 nanoparticle (B) 0.5%, (C) 1%, (D) 3%, and (E) 5%.

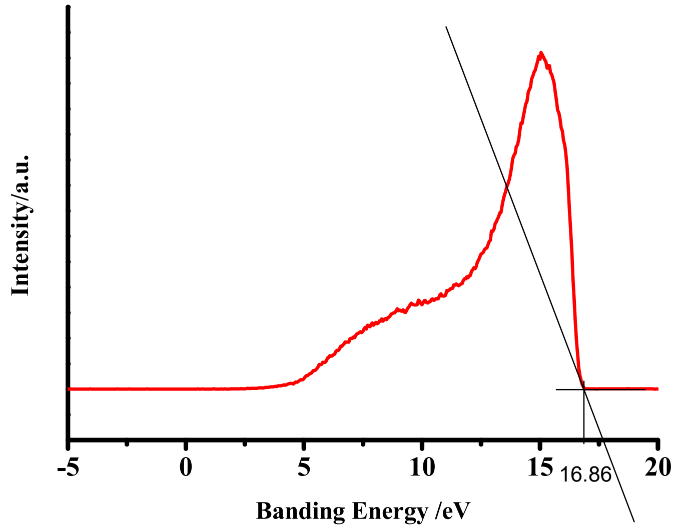

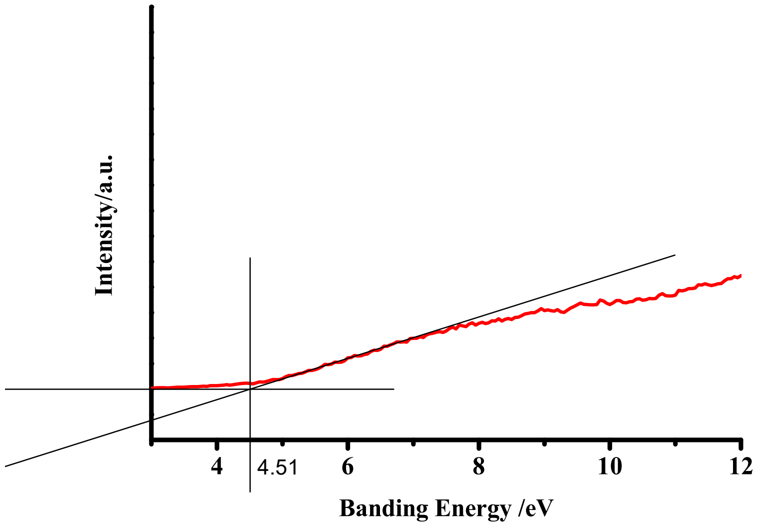

Figure S3. The ultraviolet photoelectron spectroscopy (UPS) of Zn-ZrO_2 (1%) nanoparticles.

Through the formula: WF=hν-ΔE. According to Figure S3, the work function of Zn-ZrO_2 is 4.36 eV and the VB of Zn-ZrO_2 is situated at 8.87 eV. As the S2 shows, the UV-vis absorption band located at 433 nm. Thus, the band gap between VB and CB is 2.86 eV. The CB of Zn-ZrO_2 is situated at 6.01 eV.

Table S1 Enhancement factor of 4-MBA adsorbed on ZrO_2 and Zn-ZrO_2 NPs (0.5%, 1%, 3%, and 5%).

Zn-ZrO_2 /n%	0	0.5	1	3	5
EF	4.32×10^3	1.34×10^4	1.94×10^4	1.57×10^4	1.56×10^4
